# Distinct Gut Microbiota Profiles Reflect Severity in Chronic Insomnia Disorder

**DOI:** 10.1002/brb3.71155

**Published:** 2025-12-31

**Authors:** Yaxi Liu, Yixian Cai, Xian Shi, Mei Fan, Xiaotao Zhang, Jingjing Lin, Xiaoxuan Fan, Bingdong Liu, Jiyang Pan

**Affiliations:** ^1^ Sleep Medicine Center The First Affiliated Hospital of Jinan University Guangzhou China; ^2^ Department of Psychiatry The Third Affiliated Hospital of Sun Yat‐Sen University Guangzhou China; ^3^ Department of Psychiatry, The First Affiliated Hospital of USTC, Division of Life Sciences and Medicine University of Science and Technology of China Hefei China; ^4^ Department of Endocrinology and Metabolism Zhujiang Hospital of Southern Medical University Guangzhou China; ^5^ State Key Laboratory of Applied Microbiology Southern China, Guangdong Provincial Key Laboratory of Microbial Culture Collection and Application, Guangdong Open Laboratory of Applied Microbiology, Institute of Microbiology Guangdong Academy of Sciences Guangzhou China

## Abstract

**Introduction:**

Severe insomnia symptoms increase the risk of persistent sleep disorders, underscoring the need for timely identification to optimize therapeutic interventions. Given the established association between chronic insomnia disorder (CID) and gut microbiota, this study aimed to evaluate the utility of gut microbiota characteristics for stratifying CID severity.

**Methods:**

A total of 65 patients with CID were categorized into two groups based on Pittsburgh Sleep Quality Index (PSQI) scores: S‐CID (more severe poor sleep quality; median age: 36, IQR: 30–47; M/F: 12/22) and M‐CID (milder poor sleep quality; median age: 33, IQR: 25–43; M/F: 11/20). Thirty healthy controls (HC; median age: 32, IQR: 26–48; M/F: 8/22) were also included. All participants underwent polysomnography and clinical assessments. Fecal samples were collected and analyzed via 16S rRNA gene sequencing. We compared microbial structure across severity groups, identified key bacterial genera using LASSO regression and the Boruta algorithm, and examined their correlations with sleep parameters via Spearman analysis. Functional pathway predictions were performed with PICRUSt2. A random forest model was constructed to evaluate severity‐stratified discriminative capacity.

**Results:**

Significant alterations in gut microbial diversity and composition were observed in S‐CID patients compared to HC, whereas M‐CID patients showed less pronounced differences. Seven key bacterial genera were identified and consistently correlated with sleep parameters. Functional perturbations in glutamate/butanoate metabolism and branched‐chain amino acid degradation pathways differed by severity. The random forest model demonstrated moderate efficacy (AUC = 0.711–0.730) in distinguishing S‐CID patients based on microbial signatures.

**Conclusion:**

This study reveals distinct gut microbial signatures associated with varying severity levels of CID, providing insights that may support the development of microbiota‐based diagnostic and therapeutic interventions.

## Graphical abstract

The authors used 16S rRNA sequencing and PICRUSt2 analysis to explore the link between sleep quality and gut microbiota in chronic insomnia disorder (CID) patients. Those with more severe sleep disturbances exhibited pronounced gut microbiota dysbiosis (structural/functional alterations). Specific microbial profiles showed moderate efficacy in differentiating patients with poorer sleep quality.

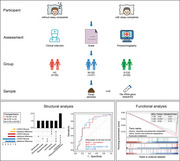

## 1 Introduction

Insomnia disorder is the most prevalent sleep disorder, characterized by difficulty initiating or maintaining sleep despite having sufficient sleep opportunities, leading to daytime functional impairment (Perlis et al. 2022). The global prevalence of insomnia disorder in adults ranges from 10% to 20%, with approximately 50% of cases following a chronic course (Morin et al. 2006; Morin and Benca 2012). Despite the high prevalence and significant disease burden, insomnia is often under‐recognized and inadequately treated (Morin et al. 2006; Morin and Benca 2012), which constitutes a pressing clinical challenge.

Currently, the diagnosis of insomnia disorder primarily relies on patients’ subjective reports rather than objective sleep measurements (Morin et al. 2015), potentially leading to clinical assessment biases. A 10‐year prospective study found that the remission rate for individuals with severe insomnia symptoms was only 56% over the course of a decade (Janson et al. 2001). The severity of insomnia may serve as an important predictor of the risk for disease progression, with more severe insomnia symptoms being associated with an increased risk of persistent sleep disturbances (Morin et al. 2020). Therefore, timely and accurate identification of patients with severe insomnia is crucial for optimizing treatment management and improving outcomes. Furthermore, despite advances in both pharmacological and non‐pharmacological treatments for insomnia, significant individual variability in clinical practice remains, resulting in the absence of standardized treatment protocols for different patients (Yue et al. 2023), which increases the uncertainty in treatment decision‐making. This highlights the urgent need for the exploration of novel, personalized therapeutic approaches.

Since the introduction of the microbiome‐gut‐brain axis (MGBA), gut microbiota have been recognized for their significant role in maintaining human health (Gilbert et al. 2016; Qin et al. 2010; Sonnenburg and Bäckhed 2016) and are closely linked to an increased risk of various diseases in the host (Martin et al. 2018). Recent years have seen growing recognition of the bidirectional nature of the MGBA, which may serve as the basis for the interaction between sleep and the gut microbiome (Wang et al. 2022). Animal studies have shown that chronic sleep fragmentation exacerbates gut microbiota dysbiosis in mice (Poroyko et al. 2016), while depletion of the gut microbiome negatively affects sleep structure (Ogawa et al. 2020). Clinical studies have found that sleep loss significantly affects the composition of the human gut microbiome, with changes in the *Firmicutes* to *Bacteroidetes* (F/B) ratio and the abundances of several gut microbiota species (Benedict et al. 2016). Critically, alterations in gut microbiota have also been linked to insomnia disorder: Liu et al. first reported that patients with chronic insomnia disorder (CID), defined as those with a disease duration of at least three months, have significant deviations in the structure and function of their gut microbiota compared to healthy individuals (Liu et al. 2019). Another study similarly found gut microbiota changes in both chronic and acute insomnia disorder (defined as those with a disease duration of less than three months), with specific bacterial signatures emerging as important biomarkers for identifying insomnia disorder and showing a significant correlation with sleep quality (Li et al. 2020). Additionally, in elderly patients with insomnia disorder, objective sleep efficiency was a significant predictor of variations in microbiota composition, with sleep quality being associated with the abundance of specific bacterial taxa (Haimov et al. 2022). A cohort study also found differences in gut microbiota characteristics between general populations with different sleep quality, with a more stable bacterial interaction pattern observed in those with good sleep quality, suggesting a more consistent co‐occurrence in this group (Seong et al. 2024). Collectively, these findings establish a clear association between gut microbiota dysbiosis and insomnia disorder, suggesting distinct microbial profiles may underlie clinical subtypes. This underscores the need for stratified research across different CID severity levels to identify characteristic microbiota features. Such features could serve as objective indicators for detecting severe cases, offering significant clinical value and informing personalized treatment strategies. However, despite this potential, a critical gap remains: existing studies have largely neglected stratification based on clinically defined severity. Consequently, the specific gut microbiota signatures associated with varying degrees of insomnia severity are poorly characterized. Furthermore, while microbiota‐targeted interventions show promise for sleep disorders (Li et al. 2023; Mudaliar et al. 2024), the evidence remains limited (Gil‐Hernández et al. 2023) and sometimes conflicting (Ho et al. 2021; Wu et al. 2021), particularly in severity‐stratified CID cohorts.

Therefore, to address this need, our study aimed to (1) characterize gut microbiota structural variations across CID patients with severity‐based stratification using 16S rRNA gene sequencing. (2) Infer microbial metabolic pathways associated with CID severity through PICRUSt2 analysis (Douglas et al. 2020). (3) Evaluate the discriminative capacity of microbiota features for clinical severity subgroups. These investigations seek to provide mechanistic insights for future therapeutic strategies in CID management.

## 2 Materials and Methods

### 2.1 Study Design and Participants

This study employed a case‐control design. All participants underwent clinical assessments and diagnoses, independently conducted by two attending psychiatrists from the First Affiliated Hospital of Jinan University. Inclusion criteria for CID patients: (1) Han Chinese ethnicity; (2) age between 18 and 65 years; (3) met the diagnostic criteria for CID according to the International Classification of Sleep Disorders (ICSD‐3); (4) Pittsburgh Sleep Quality Index (PSQI) score > 5 and Insomnia Severity Index (ISI) score > 7. Exclusion criteria for CID patients: (1) a history of other sleep disorders, mental illnesses, or severe physical conditions; (2) body mass index (BMI) ≤ 18.5 kg/m^2^ or BMI ≥ 25 kg/m^2^; (3) periodic limb movements (PLM) during sleep associated with arousals ≥ 15 or an apnea‐hypopnea index (AHI) ≥ 15 on diagnostic polysomnography (PSG); (4) Use of any medications or supplements known to affect sleep within the two weeks prior to the study, including but not limited to: benzodiazepines, Z‐drugs (e.g., Zolpidem), sedating antidepressants (e.g., Trazodone), antipsychotics (e.g., Quetiapine), antihistamines, orexin receptor antagonists (e.g., Suworexine), melatonin receptor agonists (e.g., Ramelteon), as well as other supplements or hormone‐related medications (e.g., melatonin or fish oil); (5) specific dietary habits (e.g., vegetarianism or traditional ethnic diets); (6) recent surgery or use of antibiotics or probiotics within the past two months; (7) significant life events or changes in living environment in the past six months; (8) Pregnant or lactating women.

The PSQI is one of the most widely used questionnaires for assessing sleep disorders, demonstrating high sensitivity and specificity (Buysse et al. 1989). A total score > 5 indicates poor sleep quality (Morin et al. 2015). In this study, a cutoff score of > 10 was adopted based on evidence indicating its improved efficacy in identifying clinically significant sleep impairment (Okun et al. 2009). This threshold has also been employed in multiple studies to differentiate between mild and more severe sleep disturbances (Decrinis et al. 2025; Reinsel et al. 2015; Xie et al. 2025). All CID patients included in this study had a PSQI total score > 5. Utilizing the aforementioned cutoff, we defined the M‐CID group as having milder poor sleep quality (PSQI 6–10) and the S‐CID group as having more severe poor sleep quality (PSQI > 10).

Matched health controls (HC) were socially recruited individuals unrelated to the patients. The inclusion criteria for the HC group were (1) Han Chinese ethnicity, (2) age between 18 and 65 years, and (3) PSQI score ≤ 5 and ISI score ≤ 7. The exclusion criteria for the HC group were identical to those for the CID group.

The study was approved by the Medical Ethics Committee of the First Affiliated Hospital of Jinan University (No. KY‐2022‐167). All participants provided written informed consent prior to participation, and the researchers adhered to the principles outlined in the Declaration of Helsinki.

### 2.2 Clinical Assessments and Sample Collections

All participants underwent both subjective and objective sleep measurements, along with emotional scale assessments. In addition to PSQI, ISI was employed to assess the degree of sleep difficulties and their impact on daytime functioning, with a score of seven or less indicating the absence of clinical insomnia (Morin et al. 2015). Additionally, the 17‐item Hamilton Depression Rating Scale (HAMD‐17) and the Hamilton Anxiety Rating Scale (HAMA) were used to assess depressive and anxiety symptoms experienced over the past week (Hamilton 1959, 1960).

All participants maintained their usual bedtime and wake‐up routines and underwent two consecutive nights of PSG to rule out other sleep disorders and first‐night effects. Data from the second night of PSG were analyzed in this study. The PSG results were interpreted following the standards outlined in the American Academy of Sleep Medicine (AASM) Manual for the Scoring of Sleep and Associated Events, version 2.6. The objective sleep parameters analyzed in this study included total sleep time (TST), sleep efficiency (SE), wake after sleep onset (WASO), sleep latency (SL), the total duration and proportion of each sleep stage (Wake stage [W], N1, N2, N3, Rapid Eye Movement stage [REM]), and the frequency and index of awakenings (AI) during REM, Non‐Rapid Eye Movement (NREM), and other sleep stages across the entire night.

Fecal samples were collected from all participants the morning after the second night of PSG and promptly stored in sterile cryovials at −80°C for subsequent processing.

### 2.3 16S rRNA Amplicon Sequencing and Analysis

The 16S rRNA gene sequencing data were obtained from all participants in the study. Bacterial DNA was extracted from fecal material using the GHFDE100 (Zhejiang Hangzhou Equipment Preparation 20190952, China) DNA isolation kit, following the manufacturer's instructions. The V4 region of the bacterial 16S rRNA gene was amplified by PCR using primer 515F (5′‐ GTGCCAGCMGCCGCGGTAA ‐3′) and reverse primer 806R (5′‐ GGACTACHVGGGTWTCTAAT‐3′). High‐quality amplicon sequence variants (ASVs) were obtained based on the Unoise2 algorithm and the Greengenes database, and relative abundance was calculated at the phylum, class, order, family, genus, and species levels to perform taxonomic profile analysis. Downstream bioinformatics analyses were performed using the following R packages: EasyMultiProfiler (version 0.2.7) (Liu et al. 2025) and EasyMicroPlot (version 0.5.1.25) (Liu et al. 2021), with core bacterial taxa defined as those having a relative abundance greater than 0.001 and present in over 70% of samples within each group (Liu et al. 2019).

### 2.4 Statistical Analysis

Statistical analyses of all demographic and clinical data were performed using R 4.4.1, withthe statistical significance threshold set at *p* < 0.05. Normally distributed continuous variables were expressed as mean ± standard deviation, and comparisons among three groups were conducted using analysis of variance (ANOVA), followed by the post‐hoc Least Significant Difference (LSD) test. Non‐normally distributed continuous variables were expressed as the median (P25, P75), and comparisons among three groups were performed using the Kruskal–Wallis test, followed by the post‐hoc Nemenyi test. Categorical variables were summarized using frequency (percentage) and analyzed with the chi‐square test. Correlation analysis was performed using Spearman correlation.

## 3 Results

### 3.1 Clinical Characteristics of the Participants

**FIGURE 1:**
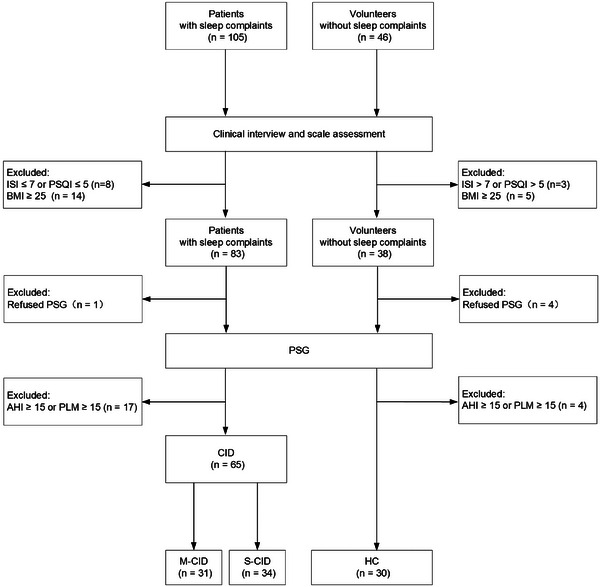
Flowchart.

Fifty‐six of the original 151 participants were excluded from the analysis, resulting in a final study sample of 95 participants (Figure 1). The participant distribution was as follows: 31 in the M‐CID group, 34 in the S‐CID group, and 30 in the HC group. As shown in Table 1, the demographic characteristics were comparable across all three groups, with no significant differences. However, significant differences were observed between the groups in several scale assessments, including PSQI, ISI, HAMA, and HAMD. Several objective sleep parameters, including SE, WASO, W, R, R%, and AIREM, also revealed significant group differences. Additionally, notable distinctions were observed between the M‐CID and HC groups, the S‐CID and HC groups, and the S‐CID and M‐CID groups.

**TABLE 1:** Comparison of general demographic and clinical data among the HC, M‐CID, and S‐CID groups.

Variable	HC (*n* = 30)	M‐CID (*n* = 31)	S‐CID (*n* = 34)	*F*/*Z*/*X* ^2^	*p*‐value
Age (year)	32 (26, 48)	33 (25, 43)	36 (30, 47)	2.68	0.262
Gender (%)				0.71	0.701
Male	8 (26.67%)	11 (31.48%)	12 (35.29%)		
Female	22 (73.33%)	20 (64.52%)	22 (64.71%)		
Female proportion (%)				5.36	0.069
Age > 50 years	0 (0%)	3 (15%)	5 (22.73%)		
Age ≤ 50 years	22 (100%)	17 (85%)	17 (77.27%)		
BMI (kg/m^2^)	20.72 (19.59, 22.92)	21.56 (20.65, 23.34)	21.12 (18.92, 22.28)	3.33	0.190
PSQI	3 (2, 4)^a^	9 (8, 10)^b^	13 (12, 15)^c^	84.00	< 0.001
ISI	2 (1, 4)^a^	15 (10, 17)^b^	17 (14, 20)^b^	63.37	< 0.001
HAMA	1 (0, 2)^a^	7 (4, 9)^b^	6 (5, 9)^b^	54.70	< 0.001
HAMD	1 (0, 2)^a^	7 (6, 10)^b^	7 (4, 9)^b^	56.17	< 0.001
TST (min)	401.00 (350.88, 439.00)	400.00 (351.50, 441.00)	360.00 (320.75, 426.38)	3.37	0.185
SE	0.88 (0.82, 0.92)^a^	0.83 (0.73, 0.93)^ab^	0.77 (0.62, 0.84)^b^	11.22	0.004
WASO (min)	36.00 (28.75, 80.38)^a^	72.00 (24.00, 104.50)^ab^	90.25 (51.62, 175.25)^b^	8.57	0.014
Wake_frequency	25 (21, 30)	28 (19, 37)	24 (14, 35)	1.51	0.469
Sleep_latency (min)	8.75 (4.50, 22.62)	12.50 (7.50, 20.50)	11.75 (7.38, 24.75)	2.50	0.286
REM_lantency (min)	85.75 (73.75, 130.88)	91.00 (59.50, 133.50)	88.00 (69.25, 146.25)	0.29	0.863
W (min)	57.50 (38.88, 84.88)^a^	81.00 (34.00, 126.50)^ab^	105.25 (74.50, 194.12)^b^	12.23	0.002
R (min)	85.87 ± 29.60^a^	86.29 ± 33.19^ab^	67.22 ± 31.07^c^	3.96	0.022
R%	21.54 ± 6.33^a^	21.89 ± 5.64^ab^	17.81 ± 6.45^c^	4.42	0.015
N1 (min)	45.75 (34.88, 62.00)	53.00 (31.00, 70.00)	39.50 (28.50, 48.25)	2.44	0.296
N1%	11.65 (8.30, 16.30)	13.80 (7.70, 17.70)	10.15 (7.50, 17.50)	0.41	0.813
N2 (min)	185.13 ± 35.78	178.89 ± 46.86	180.03 ± 59.16	0.14	0.868
N2%	45.95 (43.20, 51.05)	46.30 (40.00, 50.10)	52.55 (44.05, 54.95)	5.49	0.064
N3 (min)	73.32 ± 32.03	73.89 ± 32.22	64.44 ± 30.42	0.93	0.400
N3%	17.65 (11.88, 23.20)	19.40 (15.30, 24.00)	17.75 (11.00, 26.25)	0.11	0.947
REM_arousals	4 (1, 8)	8 (2, 17)	6 (2, 10)	4.54	0.103
AIREM	2.60 (0.55, 7.50)^a^	6.00 (2.40, 11.10)^ab^	5.80 (3.35, 11.10)^b^	6.84	0.033
NREM_arousals	39 (26, 56)	43 (31, 59)	38 (24, 63)	0.84	0.658
AINREM	7.95 (4.92, 10.72)	8.40 (6.00, 13.40)	8.00 (4.42, 13.07)	0.65	0.723
Total_arousals	50 ± 25	63 ± 35	52 ± 28	1.58	0.211
AI	6.95 (4.78, 9.68)	8.50 (6.00, 11.90)	8.75 (4.75, 11.50)	2.27	0.322

*Note*: The indicators with statistically significant differences between the three groups, as determined by ANOVA or Kruskal–Wallis test, were subjected to post‐hoc analysis. The same letters indicate no significant difference between the groups.

**Abbreviations**: AI, Index of Awakenings; AINREM, Index of Awakenings during NREM; AIREM, Index of Awakenings during REM; BMI, Body Mass Index; HAMA, Hamilton Anxiety Rating Scale; HAMD, Hamilton Depression Rating Scale; ISI, Insomnia Severity Index; N1, N1 Stage; N1%, N1 percentage; N2, N2 Stage; N2%, N2 percentage; N3, N3 Stage; N3%, N3 percentage; PSQI, Pittsburgh Sleep Quality Index; R, REM Stage; R%, REM percentage; SE, Sleep Efficiency; TST, Total Sleep Time; W, Wake Stage; WASO, Wake after Sleep Onset.

### 3.2 CID Patients With More Severe Poor Sleep Quality Exhibit Significant Dysbiosis in Bacterial Diversity and Composition

**FIGURE 2:**
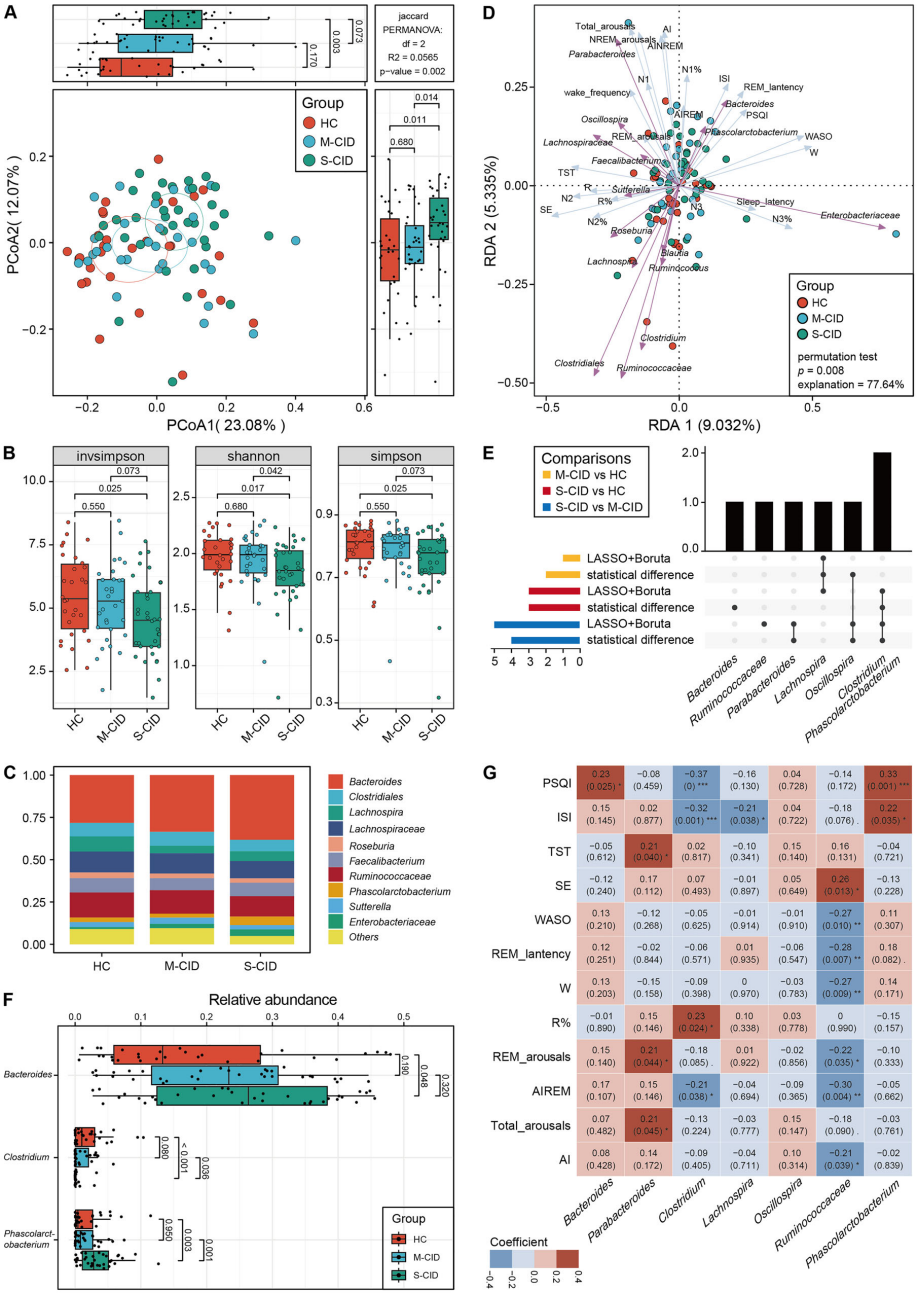
Characteristics of gut bacterial structure in the M‐CID, S‐CID and HC groups and their relationship with sleep parameters. **(A)** PCoA of β‐diversity (genus level, Binary Jaccard distance) across the three groups, **(B)** α‐diversity comparisons among the three groups, **(C)** The top ten bacterial taxa composition in the three groups, **(D)** RDA of core bacterial genera at the genus level and sleep parameters, **(E)** UpSet plot identifying key bacterial genera through three methods: LASSO regression, Boruta algorithm and difference analysis (Wilcoxon rank‐sum test). The top‐left panel defines group comparisons generating each set. Horizontal bars (left) quantify total genera per method set. Vertical bars (top) quantify genera in specific intersections. The connection matrix maps genera sharing patterns through dot‐line connections, **(F)** Box plots comparing the relative abundance of key bacterial genera among the three groups. Wilcoxon rank sum test, and **(G)** Spearman correlations between key bacterial genera and sleep parameters, with red and blue representing positive and negative correlations, respectively; ***p < 0.001, **p < 0.01, and *p < 0.05.

A total of 19 species were identified as core bacteria, which were subsequently categorized into 15 genera for further analysis (Supplementary Table S1). At the genus level, Principal Coordinate Analysis (PCoA) based on Binary Jaccard distance with Hellinger standardization and Principal Component Analysis (PCA) based on Euclidean distance with CLR standardization both indicated significant separation between the S‐CID versus HC and S‐CID versus M‐CID groups. However, no significant separation was observed between the M‐CID and HC groups (Figure 2A, Supplementary Figure S1A). The S‐CID group exhibited significantly lower values for all three α‐diversity indices compared to the HC group, and the Shannon index in the S‐CID group was also significantly lower than that in the M‐CID group (Figure 2B). The structure plot at the genus level displayed the top ten bacterial compositions across all three groups (Figure 2C). Moreover, our study revealed that the S‐CID group had a significantly lower F/B ratio compared to the HC group (Supplementary Figure S1B). Redundancy analysis (RDA) using core bacteria at the genus level demonstrated that these bacteria accounted for 77.64% of the variability in clinical data (Permutation number = 999, *p* = 0.008) (Figure 2D), suggesting a strong correlation between core bacteria and sleep parameters.

Furthermore, key bacterial taxa were identified between paired groups (M‐CID vs. HC, S‐CID vs. HC, and S‐CID vs. M‐CID) using LASSO regression and Boruta algorithms (Supplementary Table S2). Bacterial taxa exhibiting statistically significant differences between groups were also considered key taxa (Figure 2F and Supplementary Figure S1C). As shown in Figure 2E, the key genera in the comparison between the M‐CID and HC groups included *Lachnospira* and *Oscillospira*. In the comparison between S‐CID and HC groups, the key genera were *Bacteroides*, *Clostridium*, *Phascolarctobacterium*, and *Lachnospira*. Additionally, *Clostridium*, *Oscillospira*, *Parabacteroides*, *Phascolarctobacterium*, and *Ruminococcaceae* (family) were identified as key genera between the S‐CID and M‐CID groups. Notably, *Clostridium* exhibited a decreasing trend in relative abundance, with significantly higher levels in the HC and M‐CID groups relative to the S‐CID group (Figure 2F). Both *Bacteroides* and *Phascolarctobacterium* showed an increasing trend, with *Phascolarctobacterium* being significantly more abundant in the S‐CID group than in both the HC and M‐CID groups, while no significant differences were observed between the HC and M‐CID groups. The relative abundance of *Bacteroides* was significantly higher in the S‐CID group than in the HC group, with no statistical differences observed between the M‐CID versus HC groups or S‐CID versus M‐CID groups (Figure 2F). However, *Parabacteroides*, *Oscillospira*, *Lachnospira*, and *Ruminococcaceae* did not show any trend of increase or decrease across the three groups (Supplementary Figure S1C). Spearman correlation analysis was performed to examine the relationships between the key genera and sleep parameters across the entire population (Figure 2G, Supplementary Figure. S1D). The results revealed that all the key genera, except for *Lachnospira*, exhibited significant correlations with multiple sleep parameters. Notably, *Clostridium* and *Ruminococcaceae* exhibited significant negative correlations with the AIREM.

### 3.3 CID Patients With More Severe Poor Sleep Quality Show More Pronounced Dysbiosis in Bacterial Function

**FIGURE 3:**
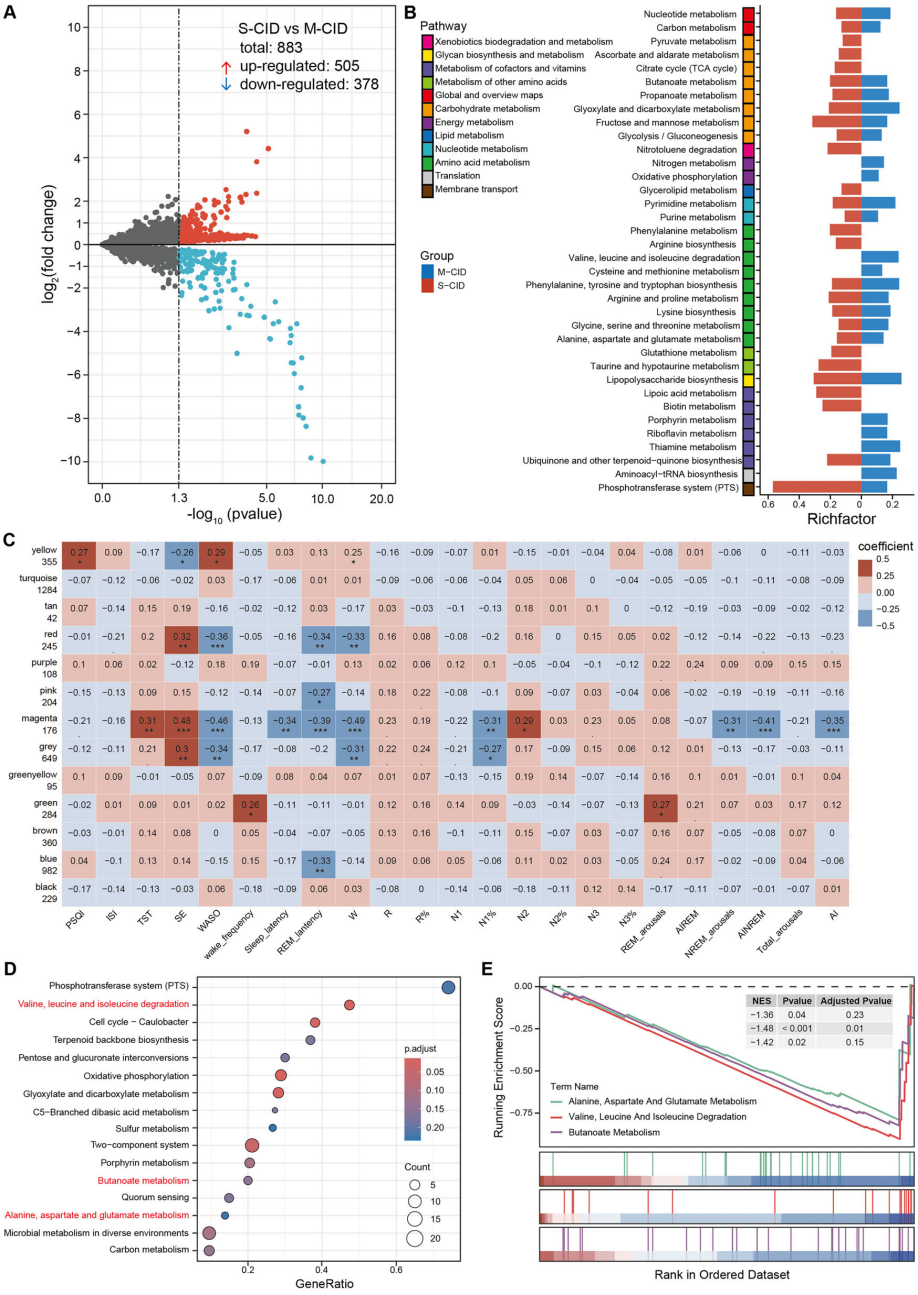
Characteristics of gut bacterial function in the M‐CID, S‐CID and HC groups and their relationship with sleep parameters. **(A)** Volcano plots of DEGs between the S‐CID and M‐CID groups. Red represents up‐regulated genes, blue represents down‐regulated genes, and gray represents genes with no significant difference, **(B)** Pathway enrichment by ORA based on DEGs, **(C)** Heatmap showing the relationships between WGCNA‐derived modules and sleep parameters. Each color represents a distinct gene module, while the gray module contains genes that could not be classified into any module. Red and blue represent positive and negative correlations, respectively. The number in each box represents the correlation coefficient. ***p < 0.001, **p < 0.01, and *p < 0.05, (D) Dot plot showing GSEA pathways enriched in CID patients. Genes ranked by log_2_FC (S‐CID vs. M‐CID). Statistically significant pathways were determined based on *p* < 0.05, adjusted *p* < 0.25 and |normalized enrichment scores (NES)| > 1, and **(E)** GSEA enrichment profile plot demonstrating non‐random distribution of gene sets.

Given potential bacterial functional redundancy, we performed PICRUSt‐based KEGG pathway prediction. Differential gene expression analysis was performed using the edgeR likelihood ratio method, focusing on the following comparisons: (1) M‐CID vs. HC, (2) S‐CID versus HC, and (3) S‐CID versus M‐CID. As shown in Supplementary Figures S2A–B, the S‐CID group exhibited a significantly greater number of differentially expressed genes (DEGs) relative to HC than the M‐CID group. Furthermore, direct comparison between S‐CID and M‐CID groups identified 883 DEGs, with 505 upregulated and 378 downregulated in the S‐CID group (Figure 3A).

Overrepresentation analysis (ORA) of the DEGs revealed that the S‐CID group showed enrichment in 50 pathways, compared to 42 in the M‐CID group (Figure 3B and Supplementary Table S3). Of these enriched pathways, 30 were common to both groups, including map00250 (Alanine, aspartate, and glutamate metabolism), map00400 (Phenylalanine, tyrosine, and tryptophan biosynthesis), map00640 (Propanoate metabolism), and map00650 (Butanoate metabolism). Notably, several of the enriched pathways have established associations with insomnia or sleep regulation (Supplementary Table S4). Collectively, the DEG profiles and ORA results suggest that functional differences between the S‐CID and HC groups were more pronounced than those between the M‐CID and HC groups.

To investigate the relationships between gut microbiota and their potential combined effects on the phenotypes of CID, we performed Weighted Gene Co‐expression Network Analysis (WGCNA) to construct a co‐occurrence network of KEGG orthologous (KO) genes. The gene modules identified through WGCNA were further correlated with sleep parameters. The results showed that KO genes were clustered into 13 modules, six of which (yellow, red, pink, magenta, blue, and green) were significantly associated with multiple sleep parameters (Figure 3C).

Gene Set Enrichment Analysis (GSEA) is a computational method used to determine whether a predefined set of genes exhibits statistically significant and concordant differences between two phenotypes. To further explore the performance of pathways enriched by KO genes in CID patients with varying sleep quality, we conducted GSEA on the genes from the identified modules that showed significant correlations with sleep parameters. As shown in Figure 3D and Supplementary Table S5, we identified 16 enriched pathways, 12 of which were downregulated in the S‐CID group, including map00280 (Valine, leucine, and isoleucine degradation), map00650 (Butanoate metabolism), and map00250 (Alanine, aspartate, and glutamate metabolism) (Figure 3E). Four pathways were upregulated in the S‐CID group.

### 3.4 Key Bacterial Taxa Can Effectively Identify CID Patients With More Severe Poor Sleep Quality

**FIGURE 4:**
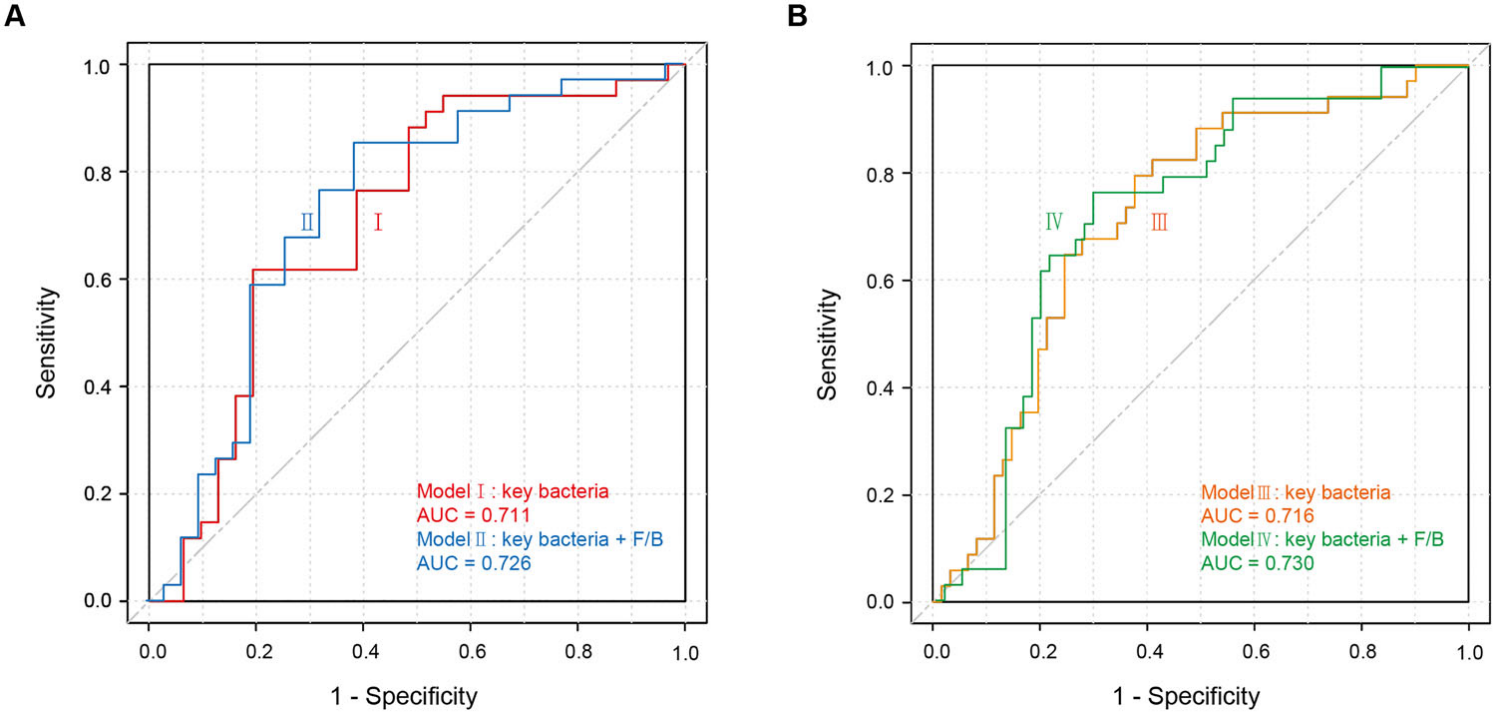
Random forest prediction model for distinguishing S‐CID patients from **(A)** CID patients or **(B)** Entire population based on gut microbial characteristics.

We applied a five‐fold cross‐validation random forest model to predict the ability of key bacterial taxa to distinguish between S‐CID and M‐CID patients. The results demonstrated that these key taxa effectively differentiated between the S‐CID and M‐CID groups (AUC = 0.711). When the F/B ratio was incorporated, the AUC increased to 0.726 (Figure 4A). Furthermore, to assess whether these key taxa could distinguish S‐CID patients from the entire population, we combined the M‐CID and HC groups into a single group (HC‐MID). The key taxa effectively differentiated between the S‐CID and HC‐MID groups (AUC = 0.716). When the F/B ratio was included, the AUC further increased to 0.730 (Figure 4B). Additional classifier performance metrics (e.g., sensitivity, specificity, and precision) were reported in Supplementary Table S6.

## 4 Discussion

This study investigated the relationship between sleep quality and gut microbial structure and function in patients with CID. We found that patients with poorer sleep quality exhibited more significant dysbiosis in terms of microbiota diversity, composition, and function. Furthermore, the random forest model demonstrated moderate discriminative capacity for more severe CID cases, suggesting microbial features may complement existing clinical assessments in severity stratification. Notably, this study employed a cross‐sectional design. Due to the inherent limitations of this approach, the findings demonstrate associations but cannot support causal inferences. Longitudinal or interventional studies are necessary to elucidate temporal dynamics. Nevertheless, our results highlight potential avenues for future research and provide preliminary evidence to support subsequent longitudinal or intervention studies targeting the gut microbiota in CID patients.

In the present study, we conducted a comprehensive comparison of gut microbial characteristics between CID patients exhibiting severe and milder poor sleep quality. Changes in bacterial diversity and composition are critical indicators of gut microbiota dysbiosis. Previous studies have reported significant alterations in both α‐ and β‐diversity in animal models of fragmented sleep (Triplett et al. 2020; Yang et al. 2023). In CID patients, significant alterations in both α‐ and β‐diversity were also found (Liu et al. 2019), and these changes were associated with the pathological stages of insomnia, with chronic insomnia showing significantly lower α‐diversity than acute insomnia (Li et al. 2020). Our study further revealed significant differences in both α‐ and β‐diversity between the S‐CID and HC groups, as well as notable differences between the S‐CID and M‐CID groups. However, no significant differences were observed between the M‐CID and HC groups. The F/B ratio is another widely used metric for evaluating gut dysbiosis and has been linked to several diseases (Wei et al. 2021). While some studies of acute sleep deprivation have reported increased F/B ratios (Benedict et al. 2016), research specifically focusing on clinical insomnia presents a distinct pattern: rodent models of insomnia show increased *Bacteroidetes* and decreased *Firmicutes* (Ren et al. 2024), and human studies have consistently reported reduced F/B ratios in CID patients (Liu et al. 2019). Our study found that a reduced F/B ratio was present only in the S‐CID group, while no significant differences were observed between the M‐CID and HC groups. This differential pattern suggests that a pronounced reduction in the F/B ratio may be a characteristic feature of more advanced disease stages rather than a universal marker of CID. Collectively, these results indicated that CID patients with poorer sleep quality exhibit more pronounced gut microbiota dysbiosis, implying that poorer sleep quality may indicate a more advanced stage of the disease, whereas milder sleep disturbances may represent a transitional phase from health to severe insomnia.

By combining two machine learning algorithms with difference analysis, our study identified seven key bacterial genera belonging to two phyla—*Firmicutes* and *Bacteroidetes*: *Clostridium*, *Ruminococcaceae*, *Lachnospira*, *Oscillospira*, and *Phascolarctobacterium* from *Firmicutes*; and *Bacteroides* and *Parabacteroides* from *Bacteroidetes*. Existing research has established that short‐chain fatty acids (SCFAs), specifically acetate, propionate, and butyrate produced by gut microbiota, are crucial for sustaining human health and can significantly impact sleep (Markowiak‐Kopeć and Śliżewska 2020; Wang et al. 2022). Notably, butyrate in particular has been implicated in beneficial regulatory effects on sleep and gut homeostasis (Hays et al. 2024; Leonel and Alvarez‐Leite 2012; Szentirmai et al. 2019), and a reduction in butyrate‐producing bacteria abundance has been noted in insomnia patients (Li et al. 2020; Wang et al. 2024). Among the identified genera, *Clostridium* is a typical butyrate‐producing bacterium that also generates acetate and propionate (Zhu et al. 2022); thus, its depletion may influence SCFA production. Supporting its relevance to insomnia, previous studies have recognized *Clostridiales* (the order containing *Clostridium*) as a key biomarker for identifying CID patients, with a significant negative correlation with PSQI scores (Liu et al. 2019). Our current study revealed a notable reduction of *Clostridium* in the S‐CID group, alongside a tendency of diminished abundance in the M‐CID group relative to the HC group. Further analysis revealed significant correlations between *Clostridium* and several subjective and objective sleep parameters, with a significant negative correlation with PSQI, ISI, and AIREM, and a significant positive correlation with R%. The correlations with R% and AIREM were particularly relevant. REM sleep‐related processes are important for subjective sleep quality (Feige et al. 2018). AIREM is a crucial index for quantifying fragmented sleep and reflects instability in REM sleep (Feige et al. 2023). Insomnia patients often exhibit increased wakefulness during REM sleep (Feige et al. 2023). As the most aroused sleep state, prolonged REM sleep may be particularly vulnerable to perception as wakefulness (Pérusse et al. 2015; Siegel 2011). Therefore, considering the reduction of *Clostridium* in CID groups, its established role in SCFA (especially butyrate) production, and its significant correlations with key REM sleep parameters, which are crucial for sleep quality, we hypothesize that *Clostridium* might influence REM sleep and sleep quality by modulating SCFA metabolism, with higher *Clostridium* abundance potentially playing a protective role in CID patients. Although the precise mechanisms linking SCFAs (especially butyrate) to sleep require further elucidation, accumulating evidence indirectly supports our hypothesis. For instance, a recent study demonstrated that fecal microbiota transplantation from insomnia patients into germ‐free mice induced insomnia‐like behaviors, which were accompanied by reduced serum butyrate levels and hyperactivity of hypothalamic orexin neurons. Importantly, intervention with tributyrin (a butyrate prodrug) suppressed orexin neuron activation and ameliorated sleep disturbances, suggesting that gut microbiota may contribute to sleep disorders through disrupted butyrate metabolism and impaired hypothalamic neuronal homeostasis (Wang et al. 2024). Furthermore, Szentirmai et al. reported that butyrate promotes NREM sleep in mice, potentially through sensory mechanisms located in the liver and/or portal vein system (2019). In a Parkinson's disease mouse model, butyrate supplementation was shown to restore normal sleep architecture, possibly via the BDNF‐TrkB signaling pathway (Duan et al. 2025).

Several studies have reported a significant reduction in *Ruminococcaceae* in patients with insomnia disorder (Benedict et al. 2016; Zhou et al. 2022), which was positively correlated with anti‐inflammatory IL‐10 and negatively correlated with PSQI and ISI scores (Zeng et al. 2024). A large cohort study found that chronic insomnia was associated with gut microbiota variations, identifying *Ruminococcaceae* UCG‐002 and UCG‐003 as potential genera inversely associated with chronic insomnia (Jiang et al. 2022). In our study, *Ruminococcaceae* was positively correlated with SE, while it was negatively correlated with WASO, AI, AIREM, etc., supporting its potential beneficial role in promoting sleep. Additionally, *Lachnospira* has been reported to inversely correlate with poor self‐reported sleep quality in acute insomnia patients (Li et al. 2020). Our study found significantly lower *Lachnospira* abundance in the M‐CID group compared to the HC group, with a significant negative correlation with ISI. *Oscillospira*, another butyrate‐producing bacterium, has been suggested as a beneficial microbe (Yang et al. 2021). Based on the Guangdong Gut Microbiome Project, a study demonstrated positive correlations between the abundance of *Oscillospira* and both microbial diversity and sleep duration (Chen et al. 2020), which were consistent with our findings. However, the association of *Phascolarctobacterium* with insomnia has been minimally studied, with one report showing its increased abundance following Traditional Chinese Medicine (TCM) treatment, inversely correlating with PSQI and ISI (Zeng et al. 2024). This contrasts with our findings, possibly due to the TCM‐specific patient inclusion criteria.

As for the *Bacteroidetes* phylum, *Bacteroides* has been identified as a key biomarker for recognizing CID (Liu et al. 2019), which was significantly elevated in CID patients and showed a positive correlation with PSQI (Liu et al. 2019). These findings were consistent with our current results. However, another study reported a decrease in the abundance of *Bacteroides* and an increase in the abundance of *Clostridium* in patients with insomnia disorder (Zhou et al. 2022), which contrasts with our findings. A potential reason for this discrepancy is that the study included both patients with chronic and acute insomnia disorder. In the core taxa identified in our study, *Parabacteroides* included only one species—*Parabacteroides distasonis*. Previous studies have shown that *Parabacteroides distasonis* was associated with a positive health status (Koh et al. 2020; Maltz et al. 2019). *Parabacteroides distasonis* may play an important role in mediating the sleep‐promoting effects of a prebiotic diet (Bowers et al. 2022). Our study found that the relative abundance of *Parabacteroides* in the M‐CID group was higher than in the S‐CID group, and it showed a significant positive correlation with TST, suggesting that *Parabacteroides* may be a potentially beneficial bacterium. In general, we observed that CID patients with poorer sleep quality had reduced beneficial bacteria and increased harmful bacteria, while those with milder insomnia symptoms showed minimal differences compared to the healthy individuals. Our analysis further confirmed significant associations between key bacterial taxa and both subjective and objective sleep parameters. However, it is important to note that while PSG provides objective sleep data, it is not optimal for CID diagnosis or severity assessment. Additionally, the lack of Multiple Sleep Latency Test (MSLT) data precluded an objective evaluation of daytime physiological sleepiness and hyperarousal. Future research should integrate MSLT‐derived measures (e.g., mean sleep latency) with subjective assessments (e.g., PSQI). This integration could facilitate subgroup analyses (e.g., comparing patients with longer vs. shorter mean sleep latency) and enable multidimensional severity stratification, thereby refining CID subtyping and enhancing our understanding of its associations with the gut microbiota.

We applied KEGG‐based PICRUSt analysis to identify the potential functional changes in the microbiota. Our results revealed that patients with poorer sleep quality exhibited more DEGs compared to healthy subjects than those with milder symptoms. Functional annotation based on DEGs revealed that the major biological functions of the enriched pathways included amino acid metabolism, carbohydrate metabolism, and SCFA metabolism, with the S‐CID group showing more pronounced enrichment. These findings suggest that alterations in amino acid, carbohydrate, and short‐chain fatty acid metabolism may occur in the gut microbiota of CID patients. Amino acid metabolism is crucial for the central nervous system, as amino acids are important sources of brain neurotransmitters and regulate neurotransmitter activity (Dalangin et al. 2020). Glutamate is the major excitatory neurotransmitter in the human brain (Weigend et al. 2019), and its signaling has a significant connection with sleep (Weigend et al. 2019), as it can be further converted into GABA (Pasanta et al. 2023), which plays a role in regulating sleep/wake cycles and promoting sleep onset (Gottesmann 2002). Although exogenous GABA is usually considered incapable of crossing the blood‐brain barrier, it may indirectly influence the nervous system through its effects on the gut (Yu et al. 2020). In the present study, we found that the alanine, aspartate, and glutamate metabolism pathway was significantly enriched in both the S‐CID and M‐CID groups. Further GSEA analysis revealed that this pathway was notably downregulated in the S‐CID group compared to the M‐CID group, indicating that it may play an important role in the regulation of sleep quality in CID. Tryptophan is the precursor to serotonin, which participates in the central nervous system's regulation of sleep/wake cycles (Brown et al. 2012; Oikonomou et al. 2019; Saper et al. 2010). Existing evidence suggests that changes in the gut microbiota's tryptophan metabolism can affect the availability of peripheral tryptophan, influencing central tryptophan levels and subsequently altering central serotonin metabolism (Gao et al. 2018, Gao et al. 2019; Lukić et al. 2019). Furthermore, CID may be associated with branched‐chain amino acid (BCAA) dysregulation, as insomnia patients exhibited elevated energy metabolism products and decreased BCAA breakdown metabolites (Gehrman et al. 2018). In our current study, we observed significant enrichment of the phenylalanine, tyrosine, and tryptophan biosynthesis pathway in both the S‐CID and M‐CID groups, while changes in the valine, leucine, and isoleucine degradation pathway were noted in the M‐CID group.

Our study also identified significant enrichment in two SCFAs metabolism pathways—butanoate metabolism and propanoate metabolism—in both the S‐CID and M‐CID groups. Notably, the S‐CID group showed a significant upregulation of the butanoate metabolism pathway compared to the M‐CID group. These pathway enrichments suggest potential alterations in the levels of the corresponding metabolites, butyrate and propionate. As previously discussed, SCFAs, particularly butyrate, have beneficial effects on insomnia disorder. Furthermore, propionate may also benefit sleep, as evidenced by an association between increased propionate concentration in human infant fecal samples and prolonged sleep duration (Heath et al. 2020). Importantly, butyrate‐producing bacteria described in the human gut are typically found in the *Firmicutes* phylum and the *Clostridiales* order, predominantly belonging to the families *Clostridiaceae*, *Eubacteriaceae*, *Lachnospiraceae*, and *Ruminococcaceae* (Fu et al. 2019). Consistent with this, most of the key taxa identified in the present study belong to *Clostridiaceae*, *Lachnospiraceae*, and *Ruminococcaceae* families. Furthermore, these key taxa showed significant correlations with multiple objective sleep parameters, especially REM sleep indicators; for instance, *Clostridium* and *Ruminococcaceae* exhibited significant negative correlations with AIREM. These findings collectively suggest functional alterations in the gut microbiota across pathological stages of CID. However, given the inherent limitations of PICRUSt2 in predicting functional potential rather than actual metabolic activity, future research should directly quantify relevant metabolites using metabolomics for validation. Targeted metabolomics, focusing on short‐chain fatty acid quantification, is planned to validate these key pathway predictions. Furthermore, integrating metagenomics for strain‐level identification and transcriptomics for gene expression dynamics will be crucial to elucidate the mechanistic links between microbial function and CID.

The random forest algorithm—a machine learning method particularly suited for high‐dimensional biological data—was employed to explore microbial signatures associated with insomnia severity. While prior studies have reported microbiota‐based classifiers for insomnia detection (Liu et al. 2019), our work extends this approach by demonstrating severity‐stratified discriminative capacity. We found that key microbial taxa showed moderate performance as potential indicators for assessing insomnia severity, and the inclusion of the F/B ratio slightly improved the model's predictive accuracy. It should be noted that, while random forest reduces overfitting risk through mechanisms such as bagging and random feature selection, this concern remains non‐negligible—particularly given the high‐dimensional yet limited sample size typical of microbiome data. The moderate AUC values observed in our model may reflect room for improvement in generalizability. To directly address overfitting concerns and enhance broader applicability, external validation in an independent cohort is necessary in future studies.

Furthermore, key confounders that may simultaneously influence both gut microbiota composition and insomnia severity—such as dietary patterns, chronic stress exposure, and circadian rhythm disruptions—were not systematically assessed in this study. Future longitudinal designs should incorporate standardized measurements of these variables (e.g., validated dietary logs, actigraphy‐derived circadian metrics, and stress biomarkers), along with covariate adjustment strategies to better isolate microbiota‐specific effects. To simultaneously address overfitting and enhance external validity, external validation in an independent cohort with matched confounder assessment is therefore warranted. Nonetheless, our findings suggest a possible biological basis for the classification of CID and support further investigation into targeted, microbiota‐based precision interventions.

## 5 Conclusion

In our study, we found that CID patients with more severe poor sleep quality exhibited disruptions in the gut microbiota, including alterations in microbial diversity, composition, and function. These disruptions were less pronounced in patients with only mild sleep quality issues. Furthermore, specific gut microbiota characteristics were able to effectively distinguish patients with poorer sleep quality. Overall, our findings provide evidence supporting the potential for using distinct gut microbiota features to identify more severe sleep quality problems. Future interventions targeting the modulation of gut microbiota composition and metabolic pathways may offer a promising approach to managing CID.

## 6 Limitations

Our study has several limitations. First, cross‐sectional design precludes causal inference regarding gut microbiota differences across groups; longitudinal studies are needed to clarify temporal dynamics. Second, 16S rRNA gene sequencing lacks strain‐level resolution; future research should integrate metagenomics, transcriptomics, and metabolomics to gain deeper functional insights. Third, although key bacteria were linked to CID severity, their functional roles require experimental validation. Fourth, the generalizability of our findings is limited to Han Chinese populations, and although the proportion of females over 50 years did not differ significantly across groups, menopausal status—a potential confounder—was not assessed. Future studies should validate these findings in ethnically diverse cohorts and explicitly evaluate menopausal status. Fifth, the absence of standardized gastrointestinal measures may have missed subclinical disturbances; future studies should incorporate these assessments.

## Author Contributions

Yaxi Liu, Yixian Cai, Xian Shi contributed equally to this work. **Yaxi Liu**: conceptualization, data curation, formal analysis, methodology, supervision, validation, visualization, writing – original draft, writing – review and editing. **Yixian Cai**: conceptualization, data curation, formal analysis, investigation, methodology, supervision, validation, writing – original draft, writing – review and editing. **Xian Shi**: conceptualization, formal analysis, methodology, validation, writing – original draft, writing – review and editing. **Mei Fan**: data curation and investigation. **Xiaotao Zhang**: investigation and methodology. **Jingjing Lin**: writing – review and editing. **Xiaoxuan Fan**: writing – review and editing. **Bingdong Liu**: conceptualization, methodology, project administration, resources, software, supervision, validation, visualization, writing – review and editing. **Jiyang Pan**: conceptualization, funding acquisition, project administration, resources, supervision, writing – review and editing.

## Funding

This work was supported by the National Key R&D Program of China (Grant No. 2022YFC2503902) to Jiyang Pan.

## Ethics Statement

This study was approved by the Ethics Committee of the first Affiliated Hospital of Jinan University (No. KY‐2022‐167).

## Consent

Informed consent was obtained from all participants.

## Conflicts of Interest

The authors declare no conflict of interest.

## Lead Contact

Further information and requests for the data should be directed to and will be fulfilled by the lead contact, Dr. Jiyang Pan (Jiypan@163.com).

## Materials Availability

This study did not generate reagents.

## Supporting information




**Supplementary Material**: brb371155‐sup‐0001‐TableS1‐S6.xlsx

**Supporting fig.1**: Characteristics of gut bacterial structure in the M‐CID, S‐CID, and HC groups and their relationship with sleep parameters. **(A)** PCA of β‐diversity (genus level, Euclidean distance) across the three groups, **(B)** Comparison of F/B ratio among the S‐CID, M‐CID, and HC groups. Wilcoxon rank‐sum test, **(C)** Box plots comparing the relative abundance of key bacterial genera among the three groups. Wilcoxon rank‐sum test, and **(D)** Spearman correlations between key bacterial genera and sleep parameters, with red and blue representing positive and negative correlations, respectively; ***p < 0.001, **p < 0.01, and *p < 0.05.

**Supporting fig.2**: Volcano plots of DEGs **(A)** Between the M‐CID and HC groups and **(B)** Between the S‐CID and HC groups. Red represents up‐regulated genes, blue represents down‐regulated genes, and gray represents genes with no significant difference.

## Data Availability

Data and images that support the findings of this study are available on request from the lead contact. This paper does not report original code. Any additional information required to reanalyze the data in this paper is available from the lead contact upon request.
