# Supplementary figures and images for: Distinct Gut Microbiota Profiles Reflect Severity in Chronic Insomnia Disorder
Source: Brain Behav. 2025 Dec 31;16(1):e71155. doi: 10.1002/brb3.71155 (PMC12755969; doi:10.1002/brb3.71155)

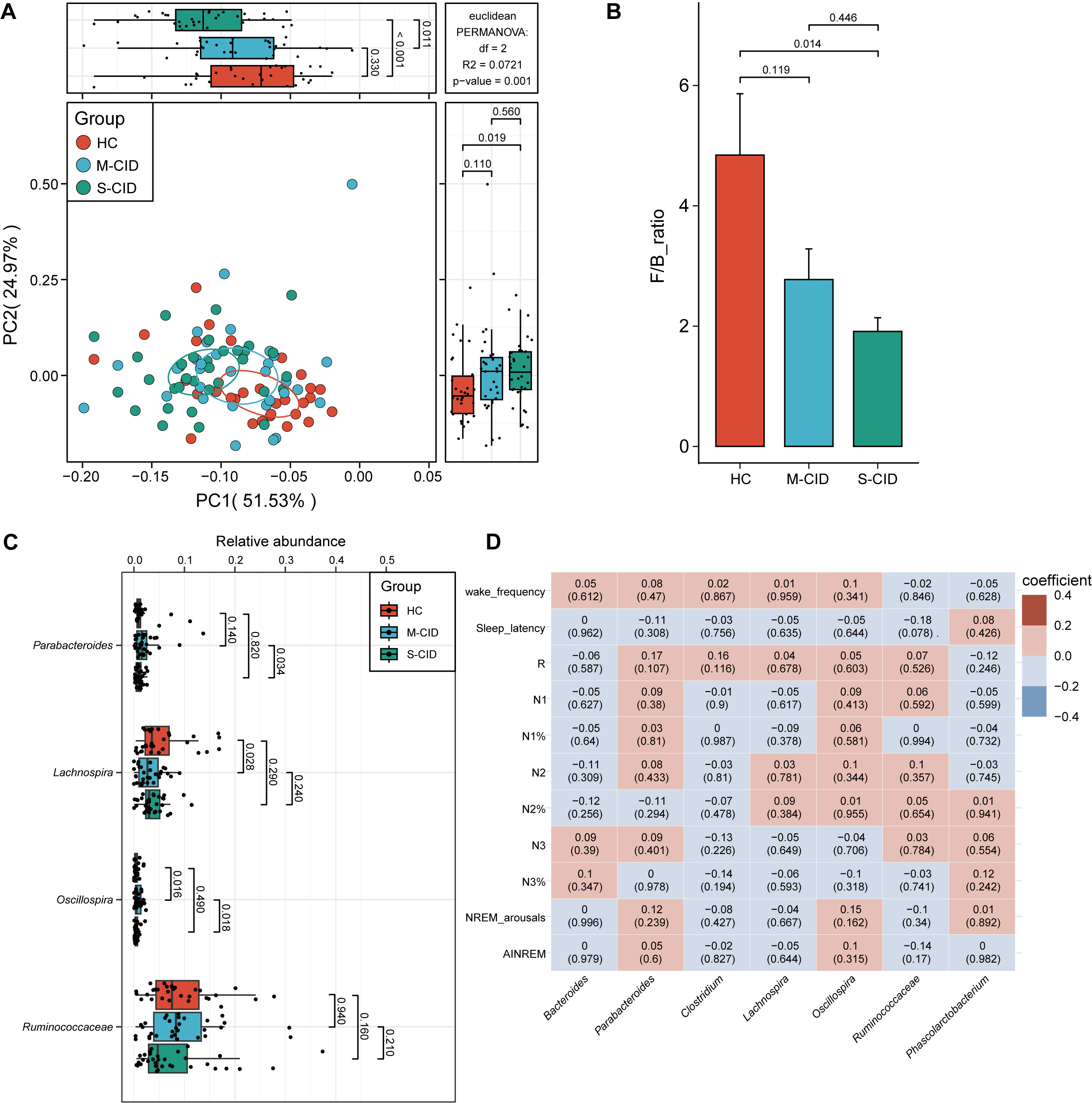

Supplement: Supplementary file 2 — Supporting fig.1: Characteristics of gut bacterial structure in the M‐CID, S‐CID, and HC groups and their relationship with sleep parameters. (A) PCA of β‐diversity (genus level, Euclidean distance) across the three groups, (B) Comparison of F/B ratio among the S‐CID, M‐CID, and HC groups. Wilcoxon rank‐sum test, (C) Box plots comparing the relative abundance of key bacterial genera among the three groups. Wilcoxon rank‐sum test, and (D) Spearman correlations between key bacterial genera and sleep parameters, with red and blue representing positive and negative correlations, respectively; ***p < 0.001, **p < 0.01, and *p < 0.05. [file BRB3-16-e71155-s002.tif]

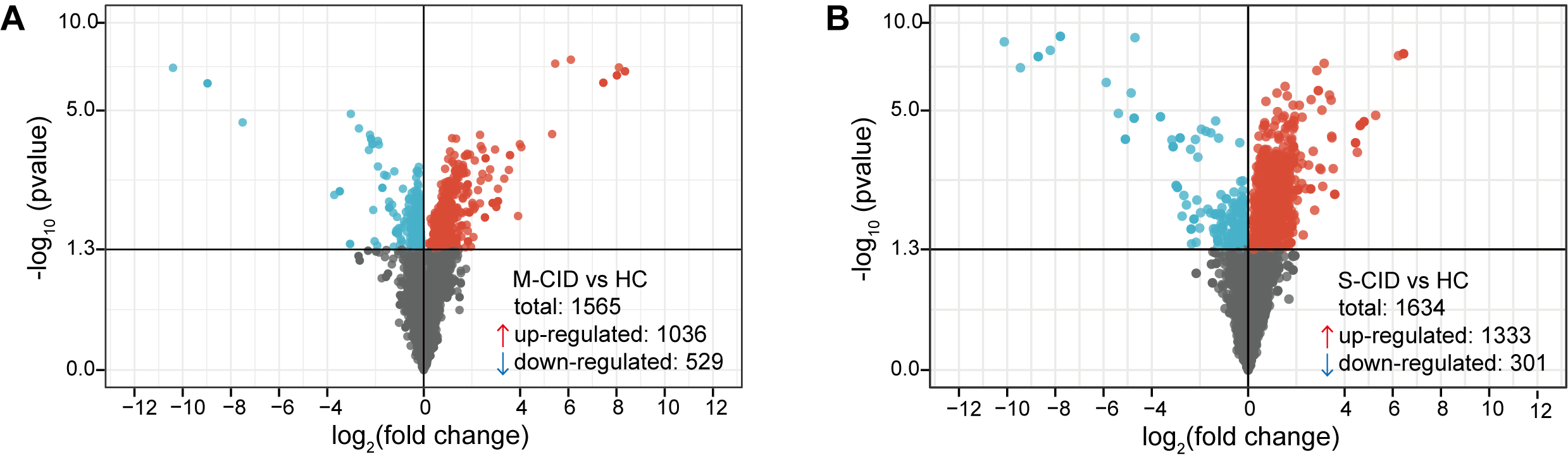

Supplement: Supplementary file 3 — Supporting fig.2: Volcano plots of DEGs (A) Between the M‐CID and HC groups and (B) Between the S‐CID and HC groups. Red represents up‐regulated genes, blue represents down‐regulated genes, and gray represents genes with no significant difference. [file BRB3-16-e71155-s003.tif]
